# Effects of Specific Virtual Reality-Based Therapy for the Rehabilitation of the Upper Limb Motor Function Post-Ictus: Randomized Controlled Trial

**DOI:** 10.3390/brainsci11050555

**Published:** 2021-04-28

**Authors:** Marta Rodríguez-Hernández, Begoña Polonio-López, Ana-Isabel Corregidor-Sánchez, José L. Martín-Conty, Alicia Mohedano-Moriano, Juan-José Criado-Álvarez

**Affiliations:** 1 Faculty of Health Sciences, University of Castilla La Mancha, 45600 Talavera de la Reina, Spain; Marta.RHernandez@uclm.es (M.R.-H.); AnaIsabel.Corregidor@uclm.es (A.-I.C.-S.); JoseLuis.MartinConty@uclm.es (J.L.M.-C.); Alicia.Mohedano@uclm.es (A.M.-M.); jjcriado@sescam.jccm.es (J.-J.C.-Á.); 2 Department of Public Health, Institute of Health Sciences, 45600 Talavera de la Reina, Spain

**Keywords:** stroke, rehabilitation, motor recovery, upper limb, virtual reality exposure therapy, occupational therapy, randomized controlled trial

## Abstract

This research analyzed the combined effect of conventional treatment and virtual reality exposure therapy on the motor function of the upper extremities in people with stroke. We designed a randomized controlled trial set in the rehabilitation and neurology departments of a hospital (Talavera de la Reina, Spain). The subjects included 43 participants, all randomized into experimental (conventional treatment + virtual reality exposure therapy) and control group (conventional treatment).; The main measures were Fugl-Meyer Assessment for upper extremity, Modified Ashworth Scale, and Stroke Impact Scale 3.0. The results included 23 patients in the experimental (62.6 ± 13.5 years) and 20 in the control group (63.6 ± 12.2 years) who completed the study. After the intervention, muscle tone diminished in both groups, more so in the experimental group (mean baseline/post-intervention: from 1.30 to 0.60; *η*^2^ = 0.237; *p* = 0.001). Difficulties in performing functional activities that implicate the upper limb also diminished. Regarding the global recovery from stroke, both groups improved scores, but the experimental group scored significantly higher than the controls (mean baseline/post-intervention: from 28.7 to 86.5; *η*^2^ = 0.633; *p* = 0.000). In conclusion, conventional rehabilitation combined with specific virtual reality seems to be more efficacious than conventional physiotherapy and occupational therapy alone in improving motor function of the upper extremities and the autonomy of survivors of stroke in activities of daily living.

## 1. Introduction

Stroke is one of the main causes of acquired disability in adulthood. The stroke epidemic is primarily driven by the aging of the world population, globalization and the urbanization of community settings [1,2]. The Stroke Alliance for Europe states that, every 20 s, a new case of stroke is detected in the adult population and predicts that the number of people affected will increase by 35% to 12 million people in 2040. As a result, it is estimated that the health and social costs for stroke diagnosis will increase to 75 million in 2030 (26% more than in 2017). In Spain, 550,941 people were diagnosed with stroke in 2017, generating a health expenditure of 1700 million euros and a total cost to the Spanish state of 3557 million euros [3].

Around 80% of survivors present motor difficulties in the upper extremities, affecting the carrying out of activities of daily living (ADLs), the performance of roles in the community and the health-related quality of life (HRQoL) [4,5,6].

Complications after stroke diagnosis can persist over time. Two-thirds of survivors are disabled 15 years later, two out of five are immersed in depressive states and more than a quarter develop cognitive impairment [7]. The costs derived from stroke diagnosis are high for survivors and their families, making their rehabilitation and survival processes a great challenge for health policymakers [8,9]. On average, an informal (non-professional) caregiver in Spain invests 2833 h per year in caring for the person affected by stroke and with limitations in ADLs [3].

The general objective of neurological rehabilitation is to promote a rapid recovery from the multiple deficits after a stroke and the achievement of a lifestyle similar to the premorbid state [10,11]. Of all people diagnosed with stroke, only 30–40% regain certain skills in the upper limb after six months of intervention [12]. The upper limb remains non-functional for ADLs in up to 66% of survivors [13], constituting the most disabling of all residual disorders.

In recent years, the use of neurorehabilitation approaches based on technology and virtual reality has increased, allowing the creation of effective rehabilitation environments and providing multimodal, controllable, and customizable stimulation [14], in which the recreation of virtual objects maximize visual feedback [15] and high intensity and high number of repetitions are key factors that influence neuroplasticity and functional improvement in patients [16]. Rehabilitation based on virtual reality offers the possibility of individualizing treatment needs, and at the same time, standardizing evaluation and training protocols [17,18]. In this sense, specific virtual reality technology for rehabilitation processes of people with neurological pathology allows working in a functional way and with specific intervention objectives, in addition to easily qualifying and documenting progress during the session [19]. Taking advantage of these characteristics, several researchers have used virtual reality exposure therapy (VRET) to recover motor function after stroke. In the treatment of the upper limb, studies indicate that this rehabilitation approach produces better motor and functional results than conventional therapy [20,21].

The increasing clinical use of neurorehabilitation approaches based on technology and virtual reality leads to the assumption that spatial representations in virtual environments may vary slightly from the perceptions that the patient would experience in real spaces. In this sense, the team of Hruby et al. [22] insisted that spatial representations based in virtual reality systems should be realistic 1:1 replicas with regard to the individual characteristics of the subjects interacting with both virtual and real environments. This demand increases the validity of virtual reality techniques for therapeutic purposes, since interaction with a virtual space is safer and more profitable in the early phases of rehabilitation processes [23]. However, it is important for clinicians and researchers to consider that the interaction with a virtual environment continues to be different from the relationship that the subject maintains with the real environment [24] because people gradually build a mental representation of the geographic space that we work with or are immersed in. The locomotion techniques applied in the virtual model (software or hardware) can influence the cognitive representations of the person experiencing them [25].

The present study aimed to analyze the combined effect of conventional treatment and VRET on motor function of the upper limb in people diagnosed with stroke in the acute phase and its evolution at three months in the Integrated Health Area of Talavera de la Reina.

## 2. Materials and Methods

### 2.1. Study Design

We began the study in April 2018 and completed the evaluation of the three-month follow-up in March 2020. The study followed the standards of the Declaration of Helsinki and was approved by the Research and Medicines Ethics Committee (CEIm) of the Integrated Area of Talavera de la Reina (protocol code: 12/2018). It is registered in the ISRCTN trial registry (ISRCTN27760662) [26].

All participants received verbal and written information about the study and gave their written informed consent.

This randomized controlled trial compared the conventional rehabilitation of physiotherapy and occupational therapy (control group) and the combination of conventional rehabilitation with the use of specific virtual reality (SVR) technology (experimental group), following the Consolidated Standards of Reporting Trials (CONSORT) guidelines [27] and CONSORT-artificial intelligence extension [28]. Change in upper limb motor skills and its impact on ADLs (baseline, post-intervention, and three-month follow-up) were used as primary outcome. The evaluation of the post-intervention variables was completed three weeks after the start of treatment in both groups (after 15 intervention sessions).

The participants were recruited from the neurology and rehabilitation units of the University General Hospital of Talavera de la Reina, Spain. They were randomly assigned to the control or experimental group by a researcher who did not participate in the intervention and the evaluation process (allocation ratio of 1:1). The conventional rehabilitation therapists were blinded to the study, but neither the participants nor the therapist who applied the VRET could be blinded to the intervention.

### 2.2. Participants and Setting

The study included 46 patients (43 of whom completed the intervention period and follow-up evaluation) who were attended in the neurology and hospital rehabilitation units (mean age 63.1, 18.6% were women) after being diagnosed with stroke. Inclusion criteria were: (1) age: 18 to 85 years; (2) maximum evolution time of six months; (3) upper limb motor involvement (Fugl-Meyer Assessment and Modified Ashworth Scale); (4) dependence in ADLs (Stroke Impact Scale, version 3.0); (5) life expectancy greater than six months (absence of life-threatening diagnoses, such as end-stage cancer); and (6) absence of other serious and disabling pathology. Four exclusion criteria were defined: presence of another neurological diagnoses, severe hemineglect, psychiatric pathology, and signature of the revocation of informed consent.

### 2.3. Outcome Measures

The primary outcome variables for this study were upper limb motor function and the impact of stroke diagnosis on ADL involving the use of the upper limb. To quantify these variables, we used the Fugl-Meyer Assessment for upper limb (FMA-UE), the Modified Ashworth Scale for the evaluation of muscle tone and the Stroke Impact Scale (SIS 3.0).

A large number of international guidelines and research in the field of neurorehabilitation and brain damage suggest that the FMA-UE is a valid instrument, given its excellent psychometric properties and its adequate scale to assess the functionality and motor function of the upper limb after a stroke. Furthermore, its use has been validated with virtual reality technology, specifically with the Kinect sensor, which is widely known and was part of the evaluation and intervention processes of this study [29,30,31,32]. The full version has 113 items, while the subscale for the evaluation of the upper limb examines 63 items (55.75%). Regarding the characteristics of the upper limb, 33 items (29.20%) evaluate motor function, 6 items (5.31%) the sensitivity and proprioception, and the last 24 points (38.09%) correspond to pain and joint mobility. Each item on the evaluation scale responds to an ordinal level of 0 to 2 points: 0 corresponds to an inability to carry out movement and 2 to a capacity to carry it out completely and adequately [29]. We applied the Spanish version of the FMA-EU [33] with a Spearman coefficient of 0.946 (*p* = 0.000) for the domain of the upper limb, excellent reliability (ICC of 0.987; *p* = 0.000), and a Cronbach’s alpha of 0.98 for motor balance of the upper limb.

The Modified Ashworth Scale measures resistance to passive movement according to a scale of 0 to 4, in which 0 corresponds to no increase in muscle tone and 4 to the affected part being rigid in flexion or extension (Kendall W of 0.765; *p* = <0.001 for elbow and a reliability of 0.4 to 0.75 for 95% of the assessments) [34].

The SIS 3.0 contains 59 items that conceptually evaluate eight important domains: strength, hand function, ADLs and instrumental activities of daily living (IADLs), mobility, communication, emotion, memory, and thinking and participation [35]. The new structure of four domains (physical, cognitive, emotional, and social participation) has conferred the SIS 3.0 a good reliability of internal consistency (Cronbach’s alpha of 0.98 for the physical domain) and test-retest (ICC of 0.79 for global recovery from stroke), concurrent validity, and responsiveness, which recommend its use in clinical practice and research [36,37,38,39].

The motor function of the upper limb, the impact of stroke on ADLs and muscle tone were evaluated and recorded before the start of treatment (baseline), at three weeks (post-intervention) and three months after its completion (follow-up). The entire evaluation process was carried out by the same researcher in both groups (an experienced occupational therapist trained for this research). In addition, we recorded sociodemographic and clinical data, such as age, sex, time elapsed since diagnosis, location of the lesion, risk factors, dominance, pain, self-perceived quality of life, or hemineglect syndrome.

### 2.4. Intervention

All study participants received 15 treatment sessions lasting 150 min per session and distributed over five consecutive days a week. In total, the intervention lasted three weeks per participant. The patients assigned to the experimental group combined conventional upper and lower limb strength and motor training (50 min physiotherapy and 50 min occupational therapy; administered by the hospital’s physiotherapy and occupational therapy team) with SVR technology devices (50 min), while participants from the control group received only conventional training in physiotherapy (75 min) and occupational therapy (75 min).

The conventional intervention protocol consisted of performing manual therapy techniques (massage), passive and active-assisted mobilizations of the upper and lower limbs, march in parallel, slope and stairs, exercises with and against resistance with balls, elastic bands and dumbbells in therapeutic cage and trellises, active-assisted mobility exercises of the upper limb and fingers in a sitting position, moving objects horizontally on the table, elevation and superposition of objects in the vertical plane, biomechanical tasks that simulated flexion-extension and abduction-adduction of the shoulder and flexion-extension of the wrist and fingers.

The motor training protocol with SVR devices consisted of the application of three systems: (1) HandTutor© glove [40,41], 3DTutor© [42], and Rehametrics© [43]. All systems are based on intensive and repetitive practice through movement instructions and feedback provided by software with virtual environments and tasks that simulate the movements that the stroke survivor requires for daily life [44,45,46].

In this work, we will address the clinical and functional effects of the use of Rehametrics© (30 min of intervention/session per participant).

The Rehametrics© software [43] and Microsoft Kinect sensor [47,48,49,50] work the upper limb (shoulder and elbow), trunk and lower body. The technology simulates ADLs and mobility in the community with virtual environments and in combination with the use of gamification. It monitors and captures the user’s movement of body, joints in real time through the Kinect sensor. In addition, the sensor calibrates the patient’s position at the beginning of each session and during exercise execution, providing visual feedback for movement and posture correction during treatment sessions. During the study, the Rehametrics© software was updated to the 2019 and 2020 versions.

The software requires the physical presence of the therapist to assess the AROM at the beginning of the session, determine the tolerance level, and adjust and customize the difficulty, duration, range of motion, and number of distracting elements or visual aids.

**Figure 1:**
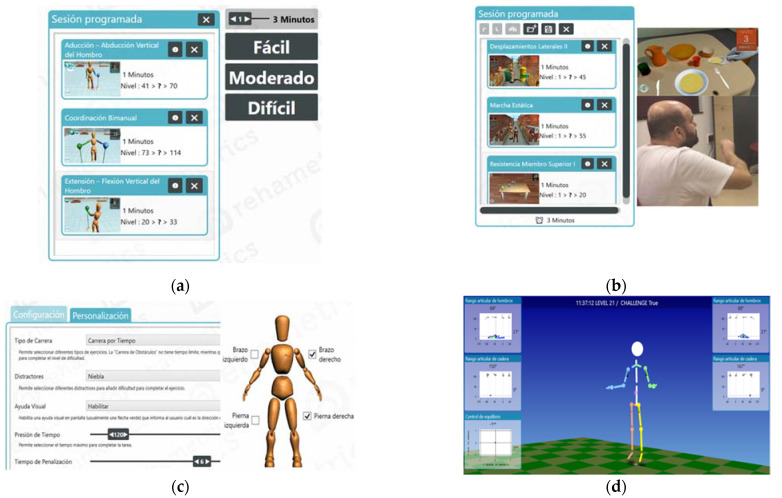
(**a**) Selection of analytical exergames; (**b**) selection of functional exergames; (**c**) adaptation of exergames at the beginning of a treatment session; (**d**) graphical representation of results or progress of the patient.

**Figure 2:**
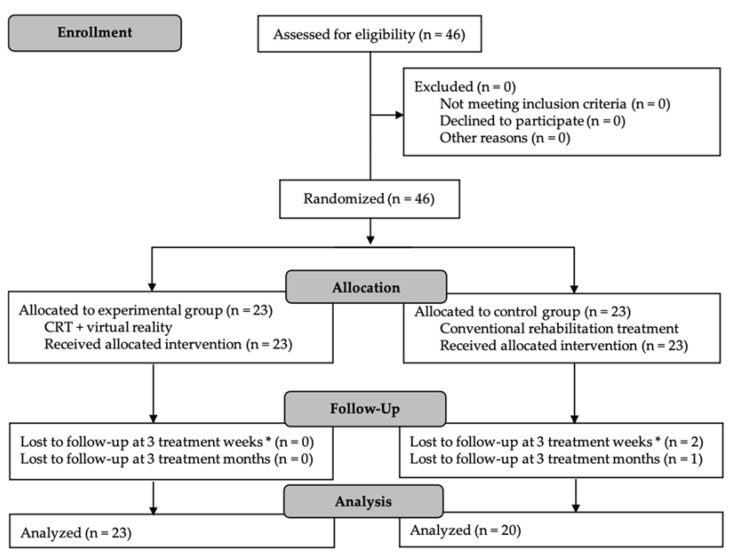
CONSORT flow diagram for participant recruitment, allocation, follow-up, and analysis. CRT: conventional rehabilitation treatment. * Post-intervention evaluation.

Rehametrics© has two types of ‘exergames’: (1) analytical exergames that work isolated movements necessary to complete an ADL-inspired flexion-extension of the elbow or abduction and adduction of the shoulder (Figure 1a) and (2) functional exergames that involve motor control, coordination of movements, contraction dynamics and displacements (Figure 1b). Before starting the treatment session, the therapist selects the exergames, the duration of each, breaks between exercises, the range of motion for each of the joints, the time pressure for the patient, the number of distractors (night or fog) and the number of visual aids (arrows that indicate if the obstacle appears on one side of the screen or another) (Figure 1c). The software automatically adjusts the level of difficulty based on the patient’s progress during each exergame. In addition, Rehametrics© provides result graphs to indicate the progress of the patient during the different treatment sessions for a given exergame, the number of failures or the ability to react to obstacles in seconds. This allows patients to visualize their progress and access a quantitative evaluation of failures, successes, and times (Figure 1d).

The exergames used were personalized according to the functional capacity of the patient, dividing them into low, medium, or high difficulty. In the first sessions, we focus on analytical exergames to increase the joint range of the upper limb. In a second phase, we selected exergames that allowed us to work in several planes and required elaborate and coordinated movements with the trunk and lower limb, lateral, and anteroposterior movements. In the final treatment sessions, elements were incorporated that allowed greater destabilization and high motor control (exergames in a sitting position on bobath balls or in a standing position on a trampoline or destabilizing base). In addition, weights were added to increase muscle strength.

Changes in active range of motion (AROM), patient position, movement correction during activity, and scheduled task execution level were extracted from Rehametrics© software and Microsoft’s Kinect sensor. These changes were not used as an outcome measure in the study. The software automatically stores these variables for each patient and exergames.

### 2.5. Statistical Analysis

The sample size was calculated with the Epidat 4.2 program. An effectiveness ratio of 90% was estimated for the experimental group and 50% for the control group. Using a power of 80% and a confidence level of 95% (*p* < 0.05), a minimum sample size of 20 participants was obtained in each group. The data were analyzed with the IBM SPSS statistical package (version 24.0) (IBM Spain, S.A., Madrid, Spain). To compare the clinical and sociodemographic variables of the groups, Student’s T and chi-square tests were used. Differences in the Ashworth, SIS 3.0 and FMA-UE scales at baseline, post-intervention, and 3-month follow-up were analyzed with inter- and intra-group ANOVA and Student’s *t* test. For the FMA-UE, a score of 7.35 in the upper limb subscale was maintained as the minimum detectable change in the three-month follow-up [33]. Statistical significance was set at a *p*-value of less than 0.05.

The analysis of missing data from the control group was carried out with multiple imputation in the analysis (expectation maximization and regression method), with a little’s chi-square statistic of 31,370 (degree freedom = 48; *p* = 0.970).

The investigator performing the statistical analysis was unaware of the random allocation of participants to the intervention groups.

## 3. Results

Forty-six patients were randomly allocated, 43 of whom completed the study period and the follow-up evaluation. Twenty-three participants were assigned to the experimental group (EG) and 23 to the control group (CG). Three participants were lost in the control group when the COVID-19 pandemic began in Spain (Figure 2).

Sociodemographic and clinical characteristics of the participants are shown in Table 1. Significant differences are observed in the evolution of pain between both groups, decreasing considerably after the intervention in the experimental group. Fifteen percent (*n* = 3) of the participants in the control group registered a change in dominance (from right to left) in the first follow-up (post-intervention), while the experimental group maintained baseline dominance. Most participants in both groups coincided in the location of the lesion in the right hemisphere (EG: 82.6% and CG: 85.0%), and more than 90% of the study participants were diagnosed with ischemic stroke (EG: 91.3% vs. CG: 90.0%). Regarding the results obtained in the visual analog scale of the EuroQoL instrument (EQ-VAS) for the self-perceived measurement of HRQoL, statistically significant differences were observed in the evolution of both groups and after the experimental intervention (EG: 86.5 vs. CG:57.0; *p* = 0.000) [51]. Rodríguez-Hernández et al. [51], authors of this study, analyzed the differences the effect of a combined treatment of conventional therapy with virtual reality on HRQoL of this sample of participants. For the analysis and its quantification, they used the following as outcome measures the EuroQoL instrument (EQ-5D-5L) and EQ-VAS.

Table 2 shows differences in the evolution of the results on the Modified Ashworth Scale between intervention groups. The decrease in muscle tone is observed in both groups, being notably higher in the experimental group (mean baseline/post-intervention: from 1.30 to 0.60). At follow-up, the muscle tone of participants in the experimental group increased slightly, while those in the control group had a more pronounced increase in muscle tone in most range of motion (mean post-intervention/follow-up: 1.05 to 1.75). The effect size of the experimental intervention was large (greater than 0.14) and statistically significant (*p* = 0.001).

The differences in the SIS 3.0 scores that reveal the impact of stroke by intervention group are shown in Table 3 and Table 4. The effect of the experimental intervention is observed in the distribution of means in both the physical dimension of force and the functional dimension of ADLs/IADLs, with statistically significant differences at baseline, post-intervention and follow-up. Exceptions were found in the evolution of strength in the most affected leg, foot, and ankle (baseline: 0.623 and 0.608; post-intervention: 0.086, respectively) and in the difficulty to reach the bathroom on time and control bladder and intestines. Marked is the decrease in the difficulty to perform functional activities that involve the use of the upper limb after the intervention in the experimental group.

**Figure 3:**
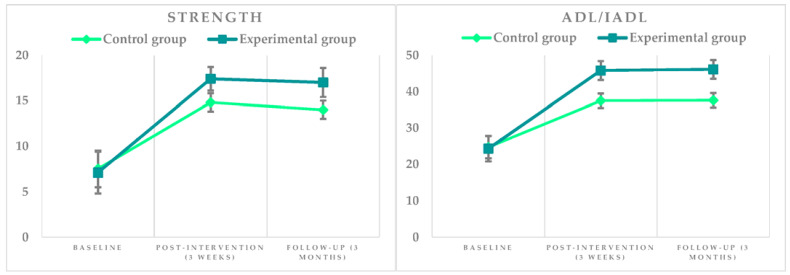
Graphic representation of the intervention’s effect on the total scores of strength domain (minimum: 4; maximum: 20) and ADLs/IADLs (minimum: 10; maximum: 50) of the SIS 3.0 instrument.

The graphic representation of the effect of the experimental intervention on the total strength scores and ADLs/IADLs (physical domain SIS 3.0) are shown in Figure 3. A progressive and positive increase is observed in the means of both groups, being more marked in the experimental group (*p* = 0.000 in post-intervention and follow-up). The means between post-intervention and follow-up do not differ in any of the groups.

Table 5 shows the differences in the evolution of the results in global stroke recovery by intervention group. The increase in SIS 3.0 score is observed in both groups, but is notably higher in the experimental group (baseline/post-intervention mean: from 28.7 to 86.5). During follow-up, the control group remained stable, while scores in the experimental group decreased slightly (mean post-intervention/follow-up: from 86.5 to 78.3). The magnitude of the effect of the experimental intervention is large (0.633) and statistically significant (*p* = 0.000).

## 4. Discussion

The results of this study suggest that VRET with Rehametrics© in combination with conventional physiotherapy and occupational therapy is effective in the rehabilitation of the upper limb in survivors of stroke in the subacute phase.

Objective and validated outcome measures were used to assess muscle tone [34], motor function of the upper limb [29], and the impact of stroke diagnosis in the physical domain and in ADLs [38], in comparison with the control group.

Several research studies have used the FMA-UE to assess the effects of the intervention on motor function in the upper limb. Their results suggest a significant difference between intervention groups, while the improvement of muscle strength has not shown significant differences before and after the intervention [17,52,53]. In our study, the increase in muscle strength in the upper limb and the differences found between the experimental and control groups can be explained by the placement of weights on the wrists during SVR training, coinciding with findings by Miclaus et al. [54].

The use of VRET in treatments leading to functional recovery of the upper limb in stroke patients is based on a new, enriched, and interactive environment that influences neuroplasticity, especially in subacute phases of the disease, leading to participants manifesting the maximum level of brain reorganization [55]. No significant differences were found in the baseline evaluation of the intervention groups, so we can assume that the results are a consequence of the use of SVR technology in active and intensive training and the demands and challenges of the exergames [54]. The use of SVR as a rehabilitation method represents an alternative therapeutic concept that is attractive to patients as they focus on the demands of the exergames and not on the repetitions or the degree of difficulty of the exercise. It facilitates the relearning of coordinated motor patterns and allows us to create an environment in which the intensity of visual and auditory feedback and training can be manipulated and systematically enhanced to create individualized motor learning paradigms. This personalization increases treatment compliance and generates a positive effect on the emotional state of patients [56].

We used the SIS 3.0 scale and physical domain subscale to examine the differences in the ability to perform ADLs after the intervention. This revealed statistically significant differences in activities involving the use of the upper limb, such as eating and dressing (*p* = 0.000). Saposnik et al. [57] examined the effects of virtual reality training in stroke patients using SIS 3.0 and reported the same significant differences as our study, despite their use of nonspecific virtual reality (NSVR). In this sense, Maier et al. [58] have recently published a meta-analysis in which they analyze the differences between NSVR and SVR in relation to motor recovery after stroke diagnosis. They conclude that studies with NSVR focus on three principles: variable practice, promotion of paretic limb use, and dosage, while more than 50% of studies with SVR include the same three principles of NSVR plus explicit and implicit feedback, increased difficulty and specific practice focused on the task. They compared the subsets of studies (NSVR vs. SVR) and observed a greater impact on motor function and activity of the upper limbs in the case of SVR with statistically significant differences.

The results of other studies coincide with the significant improvements of our study in the IADLs (shopping; *p* = 0.000). In their case, and unlike Rehametrics©, they used exergames that simulated the task of purchasing specifically [17,59]. Schuster et al. [55] explain that the differences found in the improvement of strength measured with the SIS 3.0 tool could be explained by the high number of repetitions in the movements that involve the upper limb during training with the exergames of the SVR system, which coincides with our results (*p* = 0.000).

Young-Bin et al. [60] found no statistically significant differences in the Modified Ashworth Scale after the combined intervention of virtual reality and real objects. However, in our study, the experimental group significantly decreased in muscle tone and the magnitude of the effect of the experimental intervention on this variable is large (*η*^2^ = 0.237). The differences in the findings may be due to the number of weekly sessions and the time elapsed between the diagnosis and the start of the experimental intervention.

The above results suggest that SVR exercise-based intervention programs positively affect the recovery of upper limb motor function and the ability to independently perform ADL in people with stroke.

### Limitations

The findings of the present study may not coincide with results found in stroke patients in other phases of the disease or whose onset is prolonged over time or in institutions with less intensive rehabilitation programs.

The onset of the COVID-19 pandemic hindered the monitoring of the participants. The study design initially included an evaluation of the evolution at six months since completion of the combined treatment (VRET+conventional treatment) in the experimental group or conventional therapy in the control group.

The study was limited to a single center, and the results found may differ from other multicenter studies with comparable design and methodology [61,62]. The participants could not be blinded because they agreed on the conventional rehabilitative treatment.

In addition, virtual reality software developers should expand their research to help clinicians estimate the differences that these systems have in the cognitive and spatial representations of the individual. For example, Coomer et al. [63] suggest that swinging of the upper limbs is a locomotion technique that causes a high spatial awareness in the person who experiences it through virtual reality-based technology, and other researchers argue that the teleportation in the exergames can cause spatial disorientation due to the lack of self-movement signals [64,65].

## 5. Conclusions

The conventional rehabilitation approach combined with SVR appears to be more effective than conventional physiotherapy and occupational therapy alone in improving upper limb motor function and execution and autonomy in ADL in stroke survivors. Virtual reality as a complement to conventional rehabilitation treatment is associated with a decrease in muscle tone and greater overall recovery after stroke diagnosis.

## Figures and Tables

**Table 1:** Characteristics of the participants of both groups (*n* = 43).

Characteristics: Study Variables	Experimental Group (EG)	Control Group (CG)	Difference of Mean between Groups
	(*n* = 23)	(*n* = 20)	(*p*-Value)
**Age**			−0.9 (0.812)
Mean (SD)	62.6 (13.5)	63.6 (12.2)	0.566
Under 55 years (%)	26.1	25	
55 to 70 years (%)	30.4	45	
Over 70 years (%)	43.5	30	
**Sex (%)**			
Male	78.3	85	0.571
Female	21.7	15	
**Main diagnostic (%)**			0.883
Ischemic/thrombotic	91.3	90	
Hemorrhagic	8.7	10	
**Middle cerebral artery lesion (%)**	60.9	55	0.697
**Location of the brain injury (%)**			0.832
Right	82.6	85	
Left	17.4	15	
**Time since diagnostic (days)** *			
Baseline (pre-intervention)	55.3 (34.3)	54.2 (30.4)	1.1 (0.909)
Post-intervention (3-week follow-up)	75.3 (34.3)	74.2 (30.4)	1.1 (0.909)
Follow-up (3 months)	162.3 (36.9)	157.2 (36.1)	5.1 (0.650)
**Hemispatial neglect syndrome (%)**	13	10	0.756
**Presence of pain in extremities (%)**			
Baseline (pre-intervention)	43.5	50	0.669
Post-intervention (3-week follow-up)	21.7	80	0
Follow-up (3 months)	82.6	100	0.05
**EuroQoL visual analog scale (EQ-VAS)** *			
Baseline	29.1 (12.8)	25.5 (5.1)	0.241
Post-intervention	86.5 (7.1)	57.0 (4.7)	0
Follow-up	78.3 (10.7)	58.5 (5.9)	0
**FMA-UE (Total A. Upper extremity)** *			
Baseline	12.6 (3.4)	12.7 (3.3)	−0.1 (0.930)
Post-intervention	30.1 (3.0)	24.7 (3.7)	5.4 (0.000)
Follow-up	31.1 (4.3)	26.9 (4.1)	4.2 (0.002)
**Dominance (change baseline/post-intervention) (%)**			
Right	100.0/100.0	100.0/85.0	0.054
Left	0	0/15.0	

* Mean (SD). *p*-value: Student’s *t*-test for independent samples in continuous variables/Pearson’s chi-squared test.

**Table 2:** Linear model. Effect of the intervention on Modified Ashworth Scale results.

Intervention Group				Difference Baseline/Follow-Up			
Modified Ashworth Scale (T-Score)	Baseline Mean (SD)	Post-Intervention Mean (SD)	Follow-Up Mean (SD)	Mean (CI95%)	ANOVA		
					F	*p*	*η*^2^ Parcial
Experimental group	1.30 (0.70)	0.60 (0.50)	0.87 (0.46)	0.43 (0.07/0.79) *			
Control group	1.22 (0.67)	1.05 (0.21)	1.75 (0.44)	−0.45 (−0.85/−0.04) *	12.7	0.001	0.237
*p*	0.670	0.001	0.000				

SD: Standard deviation. CI95%: 95% confidence interval. Partial *η*^2^: magnitude of effect. * The difference in means is significant at level 0.014 (experimental group) and 0.026 (control group). *p*-value: italics.

**Table 3:** Difference in means of physical domain (strength) according to intervention group as measured by SIS 3.0 (*n* = 43).

SIS 3.0. Physical Domain.	Experimental Group (EG)	Control Group (CG)	Difference of Mean between Groups
Strength	(*n* = 23)	(*n* = 20)	(*p*-Value)
1a. Of the arm that was most affected by your stroke			
Baseline	1.6 (0.7)	1.7 (0.7)	−0.04 (0.838)
Post-intervention	4.3 (0.5)	3.4 (0.5)	0.92 (0.000)
Follow-up	4.3 (0.5)	3.4 (0.5)	0.95 (0.000)
1b. Grip of your hand that was most affected by your stroke			
Baseline	1.3 (0.6)	1.4 (0.6)	−0.04 (0.800)
Post-intervention	4.2 (0.4)	3.0 (0.0)	1.22 (0.000)
Follow-up	4.3 (0.5)	3.0 (0.0)	1.30 (0.000)
1c. Of the leg that was most affected by your stroke			
Baseline	2.1 (1.0)	2.3 (0.8)	−0.13 (0.623)
Post-intervention	4.4 (0.5)	4.2 (0.4)	0.24 (0.086)
Follow-up	4.2 (0.7)	3.8 (0.4)	0.37 (0.046)
1d. Of the foot/ankle that was most affected by your stroke			
Baseline	2.0 (0.9)	2.2 (0.8)	−0.13 (0.608)
Post-intervention	4.4. (0.5)	4.2 (0.4)	0.24 (0.086)
Follow-up	4.2 (0.7)	3.8 (0.4)	0.37 (0.046)

Mean (SD). *p*-value: Student’s *t*-test for independent samples in continuous variables. Values of the SIS 3.0 (physical domain; strength): 1 (no strength at all); 2 (a little strength); 3 (some strength); 4 (quite a bit of strength) and 5 (a lot of strength).

**Table 4:** Difference in means of physical domain (ADL/IADL) according to intervention group as measured by SIS 3.0 (*n* = 43).

SIS 3.0. Physical Domain.	Experimental Group (EG)	Control Group (CG)	Difference of Mean between Groups
ADL/IADL	(*n* = 23)	(*n* = 20)	(*p*-Value)
5a. Can you cut your food with a knife and fork?			
Baseline	1.5 (0.6)	1.7 (0.6)	−0.17 (0.317)
Post-intervention	4.6 (0.5)	3.3 (0.5)	1.28 (0.000)
Follow-up	4.6 (0.5)	3.3. (0.5)	1.27 (0.000)
5b. Can you dress the top part of your body?			
Baseline	1.7 (0.8)	1.7 (0.6)	−0.04 (0.836)
Post-intervention	4.9 (0.3)	3.4 (0.5)	1.49 (0.000)
Follow-up	5.0 (0.0)	3.4 (0.5)	1.60 (0.000)
5c. Can you bathe yourself?			
Baseline	1.6 (0.7)	1.7 (0.6)	−0.09 (0.667)
Post-intervention	4.5 (0.5)	3.3 (0.5)	1.14 (0.000)
Follow-up	4.6 (0.5)	3.4 (0.5)	1.22 (0.000)
5d. Can you clip your toenails?			
Baseline	1.3 (0.5)	1.3 (0.6)	−0.09 (0.599)
Post-intervention	3.7 (0.8)	3.2 (0.4)	0.51 (0.010)
Follow-up	3.7 (0.8)	3.2 (0.4)	0.50 (0.013)
5e. Can you get to the toilet on time?			
Baseline	3.2 (1.0)	3.2 (0.8)	0.04 (0.870)
Post-intervention	4.8 (0.4)	4.5 (0.5)	0.26 (0.073)
Follow-up	4.8 (0.4)	4.6 (0.5)	0.23 (0.109)
5f. Can you control your bladder (not have an accident)?			
Baseline	5.0 (0.0)	5.0 (0.2)	0.04 (0.323)
Post-intervention	5.0 (0.0)	5.0 (0.2)	0.05 (0.301)
Follow-up	5.0 (0.0)	5.0 (0.2)	0.05 (0.289)
5g. Can you control your bowels (not have an accident)?			
Baseline	5.0 (0.0)	5.0 (0.2)	0.04 (0.323)
Post-intervention	5.0 (0.0)	5.0 (0.2)	0.05 (0.301)
Follow-up	5.0 (0.0)	5.0 (0.2)	0.05 (0.289)
5h. Can you do light household tasks/chores (e.g., dust, make the bed, take out garbage, do the dishes)?			
Baseline	1.7 (0.6)	1.7 (0.4)	0.00 (1.000)
Post-intervention	4.7 (0.5)	3.8 (0.4)	0.93 (0.000)
Follow-up	4.7 (0.5)	3.8 (0.4)	0.95 (0.000)
5i. Can you go shopping?			
Baseline	1.7 (0.5)	1.7 (0.4)	−0.09 (0.532)
Post-intervention	4.4 (0.5)	3.1 (0.4)	1.34 (0.000)
Follow-up	4.5 (0.5)	3.1 (0.4)	1.38 (0.000)
5j. Can you do heavy household chores (e.g., vacuum, laundry, or yard work)?			
Baseline	1.7 (0.5)	1.7 (0.4)	−0.09 (0.532)
Post-intervention	4.3 (0.6)	3.0 (0.4)	1.21 (0.000)
Follow-up	4.3 (0.6)	3.1 (0.4)	1.25 (0.000)

Mean (SD). *p*-value: Student’s *t*-test for independent samples in continuous variables. Values of the SIS 3.0 (physical domain; ADL/AIDL): 1 (could not do at all); 2 (very difficult); 3 (somewhat difficult); 4 (a little difficult) and 5 (not difficult at all).

**Table 5:** Linear model. Effect of the intervention on global recovery from stroke (SIS 3.0).

Intervention Group				Difference Baseline/Follow-Up			
	Baseline Mean (SD)	Post-Intervention Mean (SD)	Follow-Up Mean (SD)	Mean (CI95%)	ANOVA		
					*F*	*p*	*η^2^* Parcial
**Global Recovery SIS 3.0** **(T-Score)**							
Experimental group	28.7 (12.5)	86.5 (7.1)	78.3 (10.7)	−49.5 (−56.4/−42.8) *			
Control group	26.1 (4.5)	56.7 (4.8)	59.0 (6.4)	−33.0 (−36.8/−29.1) *	70.6	0.000	0.633
*p*	0.359	0.000	0.000				

SD: Standard deviation. CI95%: 95% confidence interval. Partial *η*^2^: magnitude of effect. * The difference in means is significant at level 0.000. *p*-value: italics.
